# Supplementary material for: The Virome of ‘Lamon Bean’: Application of MinION Sequencing to Investigate the Virus Population Associated with Symptomatic Beans in the Lamon Area, Italy
Source: Plants (Basel). 2022 Mar 15;11(6):779. doi: 10.3390/plants11060779 (PMC8951528; doi:10.3390/plants11060779)
Supplement: Supplementary file 1 [file plants-11-00779-s001.zip › plants-1599864-supplementary/Figure_S1-S2.pdf]

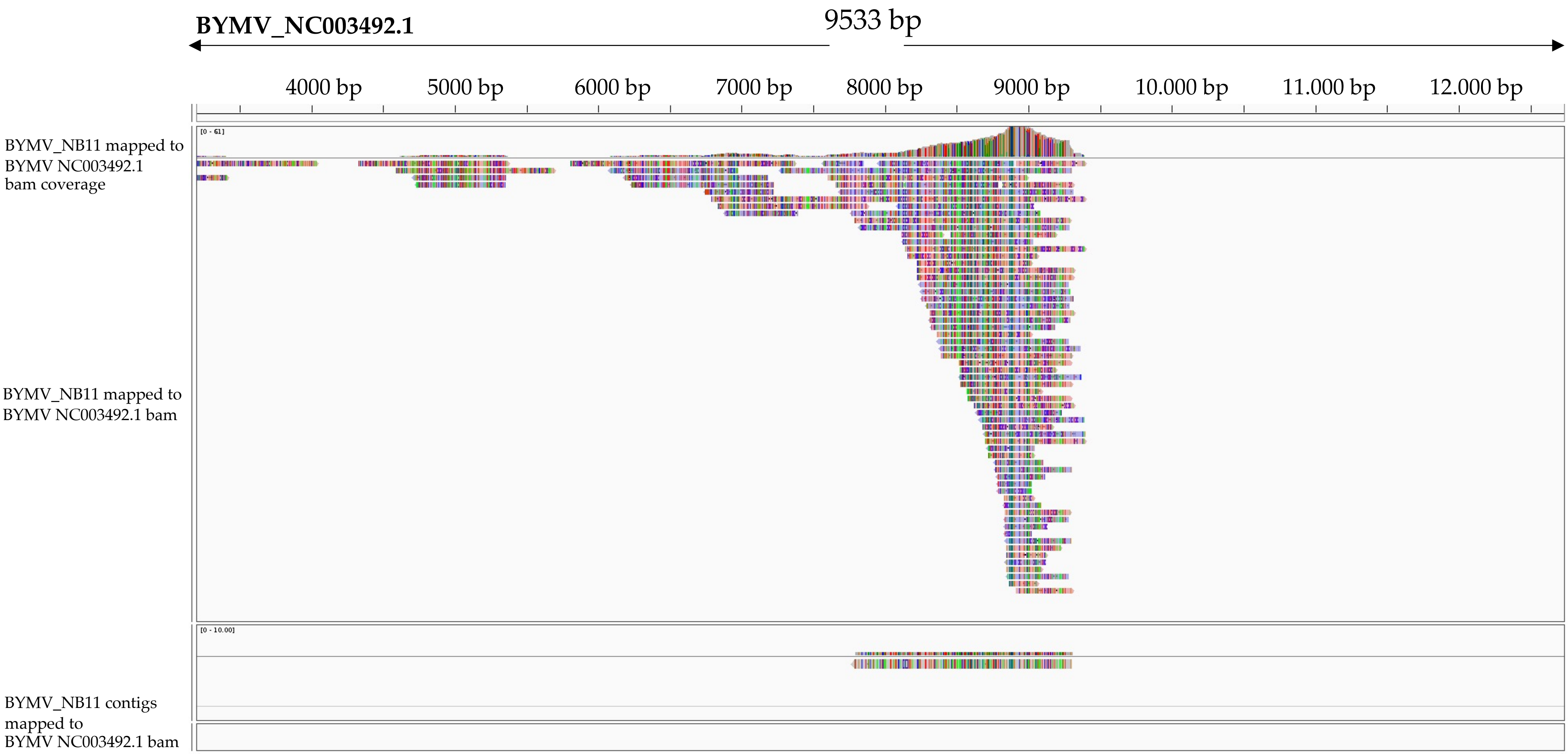

Figure S1 – Reads from sample NB11 assigned to BYMV and resulting contig. Reads from sample NB11 assigned to BYMV are aligned back to BYMV_NC003492.1 genome and visualized in IGV genome browser. A contig obtained assembling such reads is also aligned back to BYMV_NC003492.1 genome. The alignments of both reads and contig show a high number of systematic differences, suggesting biological differences between the sequenced strain and the strain available in the database, rather than sequencing errors.

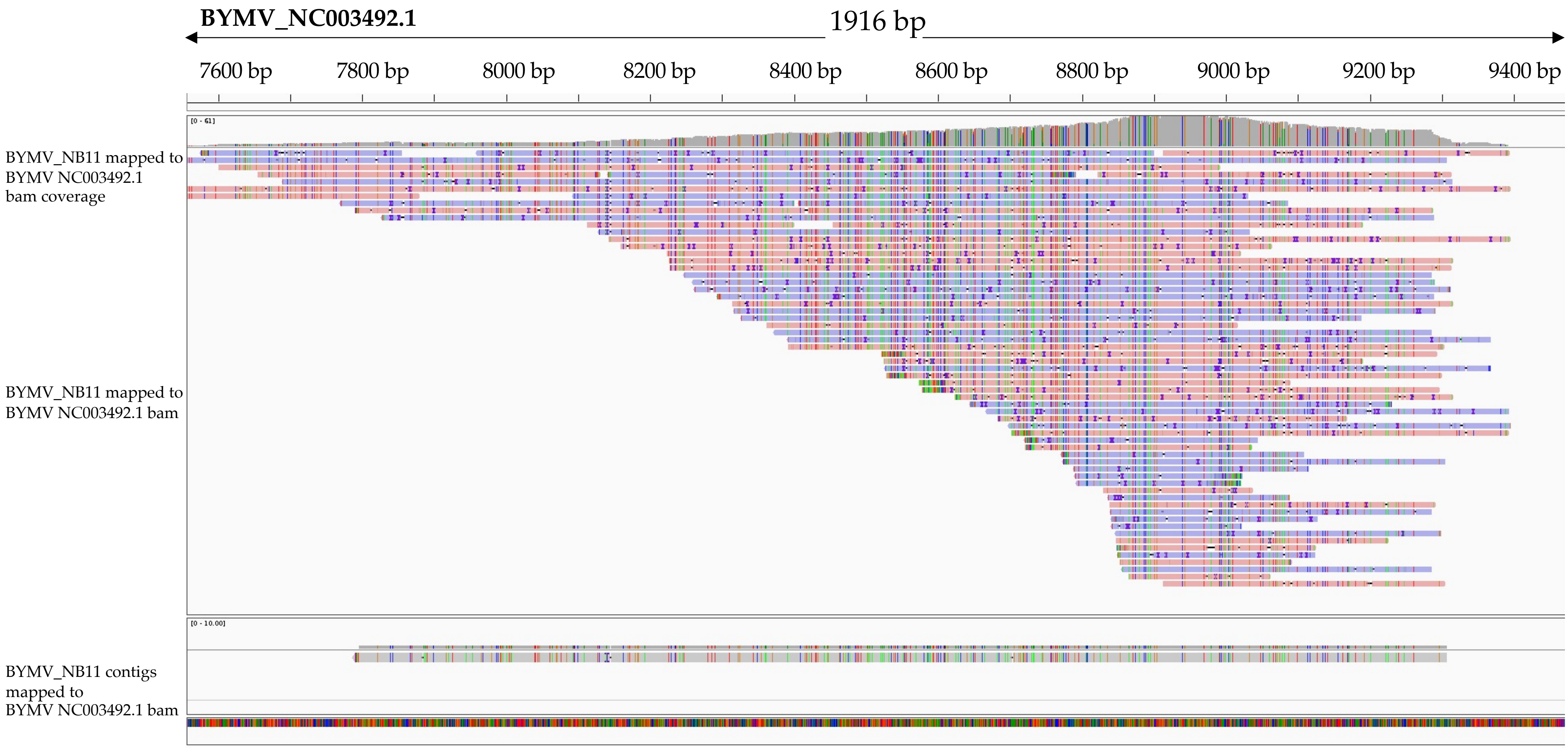

Figure S2 – Reads from sample NB11 assigned to BYMV and resulting contig, magnification.
