# Supplementary material for: The Virome of ‘Lamon Bean’: Application of MinION Sequencing to Investigate the Virus Population Associated with Symptomatic Beans in the Lamon Area, Italy
Source: Plants (Basel). 2022 Mar 15;11(6):779. doi: 10.3390/plants11060779 (PMC8951528; doi:10.3390/plants11060779)
Supplement: Supplementary file 1 [file plants-11-00779-s001.zip › plants-1599864-supplementary/Figure_S4.pdf]

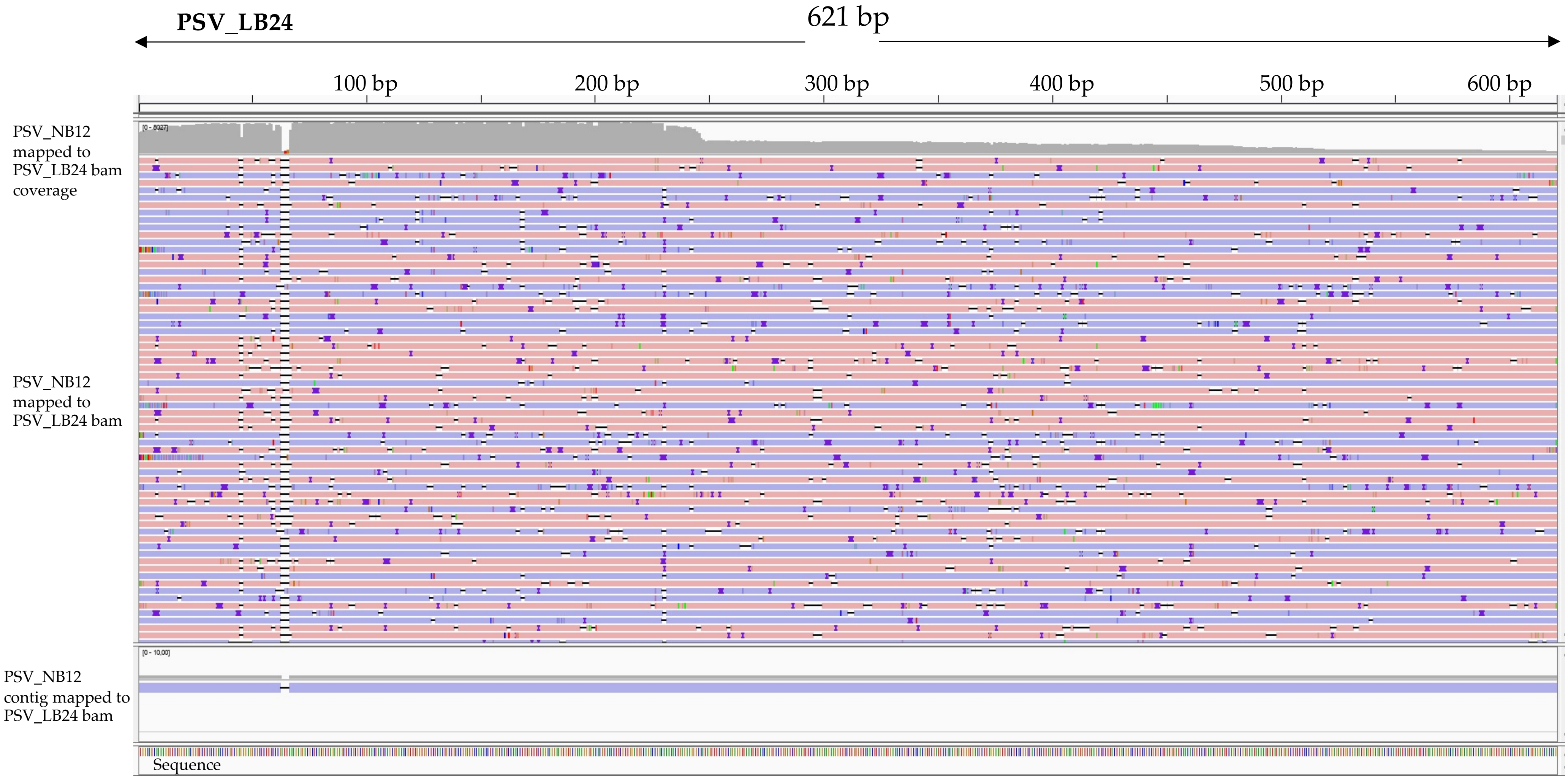

Figure S4 – Reads from sample NB12 assigned to PSV and resulting contig. Reads from sample NB12 assigned to PSV, and resulting contig, are aligned back to PSV_LB24 Sanger sequence and visualized in IGV genome browser.
